# Effectiveness of exercise interventions on muscle mass among older adults with sarcopenic obesity: A scoping review

**DOI:** 10.1002/agm2.12288

**Published:** 2024-02-06

**Authors:** S. Janani, R. Sedhunivas

**Affiliations:** ^1^ Atthi Naturopathy and Yoga Medical College Gudiyatham India; ^2^ Garden City University Bangalore India

**Keywords:** exercise interventions, impact, muscle mass, sarcopenic obesity

## Abstract

A disease known as sarcopenic obesity is characterized by decreased lean body mass in conjunction with excessive amounts of adipose tissue. Skeletal muscle mass, also known as SMM, is responsible for the largest share of fat‐free mass in the body and plays an extremely vital role in the maintaining of metabolic health. Physical activity and exercise boosts the physiological health and overall quality of life of senior citizens. The objective of the study is to investigate the effectiveness of varied exercise interventions among the geriatric people with sarcopenic obesity. During the time period (2016–2023), a scoping review was undertaken using PubMed, orthopedic journals, and the Google Scholar database, and six literature evidences relating to the topic were discovered and subsequently analyzed. The study includes six randomized control trial publications that investigated the effectiveness and impact of exercise therapies on sarcopenic obesity. According to the pre and post‐test values found in the reviewed articles, we discovered that resistance exercise is more effective than aerobic or combination exercise therapies. In conclusion, according to this scoping analysis, resistance training is more effective than other types of exercise in improving muscle mass in older people with sarcopenic obesity.

## Graphical abstract

Resistance training is the best intervention in treating the patient with sarcopenic obesity, because it directly focuses on direct muscle bulk improvement by acting on muscle fibers.

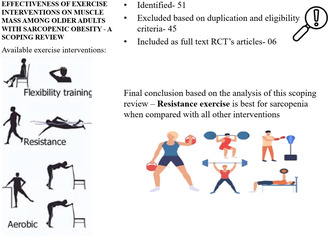

## 1 INTRODUCTION

Sarcopenic obesity is a condition characterized by diminished lean body mass in the presence of excessive adiposity. Sarcopenic obesity is most commonly documented in the elderly, as both risk and prevalence rise with age due to physiological changes in the body. Obesity worsens sarcopenia, increases fat infiltration into muscle, reduction of physical function, and raises the chance of mortality. It is now recognized as a muscle disease (muscle failure), with low muscle strength contributing to reduced muscle mass.^1^

Obesity‐mediated variables and pathways have been proposed to worsen sarcopenia and muscular functional capacity in the elderly via a local pro‐inflammatory condition.^2^ It is caused by an abnormal decrease in skeletal muscle protein synthesis and/or an increase in skeletal muscle protein degradation.^3^

The aging‐related hormonal decrease causes muscle fiber atrophy and the buildup of abdominal and intramuscular fat, predisposing the patient to sarcopenic obesity.^4^ In the elderly, sarcopenic obesity is associated with increasing body fat mass (BFM) and decreasing appendicular skeletal muscle mass (ASM). Sarcopenic obesity has been linked to established cardiovascular disease (CVD) risk variables as well as death in the geriatric population.^5^ Most chronic illnesses worsen with age, which is accompanied with major body composition changes, such as fat mass growth and redistribution and muscle and bone mass loss.^6^, ^7^

Sarcopenia was found in around 42.0% of persons over the age of 60 years. Furthermore, a two‐fold increase in the risk of death has been documented for every 1 kg/m^2^ drop in muscle mass.^3^ According to sarcopenia research, human skeletal muscle diminishes by 3%–10% per decade beginning at the age of 25.^8^, ^9^ Sarcopenia is a major illness that not only causes health issues and physical limitations, but also enormous social medical costs.^3^

Muscle volume peaks in the third decade of life, then gradually declines beginning in the fourth decade, with an 8% loss in the seventh decade and further 15% decreases per decade thereafter. Sarcopenia, on the other hand, affects 15%, 25%, 36%, and 38% of people in their 20s, 30s, 40s, and 50s, respectively.^3^ The global population of older adults (those over the age of 65 years) is growing. In 2017, older persons accounted for 13% of the world population and are anticipated to reach 2.1 billion by 2050.^6^

Technical difficulties to get an accurate measurement of muscle mass as well as muscular quality.^1^ Because body size is connected with muscle mass, either the skeletal muscle mass (SMM) or the ASM can be changed to account for body size, or through various methods, measured as by use height squared (ASM/height2).

Skeletal muscle mass accounts for the greatest proportion of total body fat‐free mass and are very important to the maintenance of metabolic health, particularly the maintenance of glucose and insulin homeostasis. Despite the fact that it has been hypothesized that adolescents who are overweight have higher levels of SMM than their counterparts who are not overweight, glucose/insulin metabolism is frequently compromised, especially in children and young adults.^2^

Appendicular lean mass and skeletal muscle index (limb muscle mass in kg divided by height in meters squared) decline with age.^10^ Importantly, the rise in fat mass and decrease in lean mass that occurs with aging may be overestimated due to fat redistribution and skeletal muscle lipid infiltration. Thigh muscle loss was identified as the strongest predictor of mortality among several metabolic outcomes studied in older persons.^11^ It is believed that by the seventh decade of life, 10%–20% of muscle mass has been lost.^6^, ^12^

Older persons lose 0.5% of their total SMM and 0.3%–4.2% of their muscle strength daily. Leg strength falls 10%–15% every 10 years until 70 years, and then 25%–40% every 10 years. Sarcopenia in older persons is caused by limited exercise, poor diet, loss of type‐II muscular fibers in skeletal muscles, and decreased blood insulin‐like growth factor 1 (IGF‐1).^13^ In terms of human health, sarcopenia increases the risk of falls and fractures, impairs ability to perform daily activities, is associated with cardiac disease, respiratory disease, and cognitive impairment, and contributes to mobility disorders, loss of independence, need for long‐term care placement, and death.^1^ Sarcopenia has traditionally been characterized and studied in geriatric populations, although new evidence reveals that younger people are also at risk.^14^

Recent research has discovered a clear link between sarcopenic obesity and physical performance decline.^15^ Muscles should be exercised as early as feasible in order to retain muscle mass and strength in old age. To maintain health for a long period, it is vital to manage health care for each life cycle, beginning with adolescence and continuing through middle age.^3^ The loss of muscle strength with age is three times quicker than the loss of muscle mass, prompting substantial research into the mechanisms underlying the age‐related decrease in muscle quality.^16^, ^17^, ^18^ In earlier studies, muscle quality was defined as muscle strength related to a certain quantity of muscle mass,^1^ with muscle strength tested using different methods (eg, one‐repetition maximum and handgrip strength).^6^

Exercise can be used as both a therapeutic and a preventative measure. Exercise training can help you gain muscular mass, strength, and protein production. Ozaki et al discovered that older persons who engage in moderately strenuous aerobic exercises on a regular basis have enhanced maximum oxygen uptake, muscle growth, and function in the lower extremities.^13^

Successful techniques that can promote fat mass loss while retaining muscle and bone mass are crucial for lowering aging‐ and obesity‐related cardiometabolic hazards, and at the same time.^6^

When compared to diet alone or exercise alone, diet with exercise induces fat mass loss while limiting weight loss‐induced loss of muscle and bone mass.^19^ Furthermore, diet with exercise resulted in the best improvement in physical function and reduction in frailty among the lifestyle interventions.^6^

Sarsan et al has shown that both aerobic training (AT) and resistance training (RT) can successfully alter obesity variables. Previous research has shown that AT has a beneficial effect on lowering obesity risk factors, but RT has resulted in increased muscle mass and strength.^13^

Exercise improves older people's physiological health and quality of life. Resistance weight training, according to,^20^ is an excellent measure for improving the health of elderly persons with sarcopenia. Resistance exercise of moderate intensity has been shown to assist older persons increase muscular size and strength.^8^, ^21^

## 2 MATERIALS AND METHODS

### 2.1 Eligibility criteria

The articles were chosen from the years 2016 to 2023. Only randomized controlled trials (RCTs) judged relevant to the topic were included from the available worldwide evidence. Guidelines, literature reviews, systematic reviews, consensus statements, and conference statements are not included in the study. The global search language was English.

### 2.2 Information source

An exclusive search approach was used to gather evidence. An exhaustive sophisticated search was conducted in PubMed, Google Scholar, and orthopedic‐related journals from 2016 to 2023.

### 2.3 Search

The scoping review was carried out between September and October 2023 utilizing the prior database search approaches, with the following keywords utilized in various combinations: “sarcopenic obesity,” “exercise training,” “skeletal muscle mass,” and “appendicular muscle mass.” Boolean logic (AND, OR, and NOT) was used to generate alternative combinations of search phrases.

### 2.4 Selection of source of evidences

In the database search, we were able to identify approximately 29 articles, of which 23 were excluded due to various reasons, such as animal trials and not focusing on the concept of exercise for patients with sarcopenic and obesity, other reviews, and thus only six relevant RCT articles were found eligible and were retrieved as full text. The co‐investigator also reviewed the collected articles. Because the review does not normally involve a human population, informed consent was not obtained. This scoping review also adhered to the Preferred Reporting Items for Systematic Reviews and Meta‐Analyses (PRISMA) guidelines checklist for scoping reviews, and eligibility criteria were constructed utilizing the Participant, Concept, and Context (PCC) framework technique.

### 2.5 Participants

To be included in this review, relevant RCTs must focus on various organized exercise regimens prescribed for older persons with sarcopenic obesity, with a focus on outcome measures, such as SMM and appendicular muscle mass.

### 2.6 Concept

The major goal of this study was to examine several forms of exercise routines that were offered as part of the care for people with sarcopenic obesity. We wanted to know how effective the multiple structured methods that were freely available were (*P* value). As a result, we opted to focus solely on RCTs that were easily available. Despite the fact that sarcopenic obesity is becoming more common worldwide, there have been very few RCTs published, indicating that researchers are less aware of and interested in the condition.

### 2.7 Context

This review was not intended to evaluate a specific geographically affected population, but rather to investigate various types of exercise available globally, with a focus on evaluating the effectiveness on SMM and appendicular muscle mass in patients with sarcopenia and obesity, which has dramatically increased among the affected global population.

### 2.8 Search strategy

**FIGURE 1:**
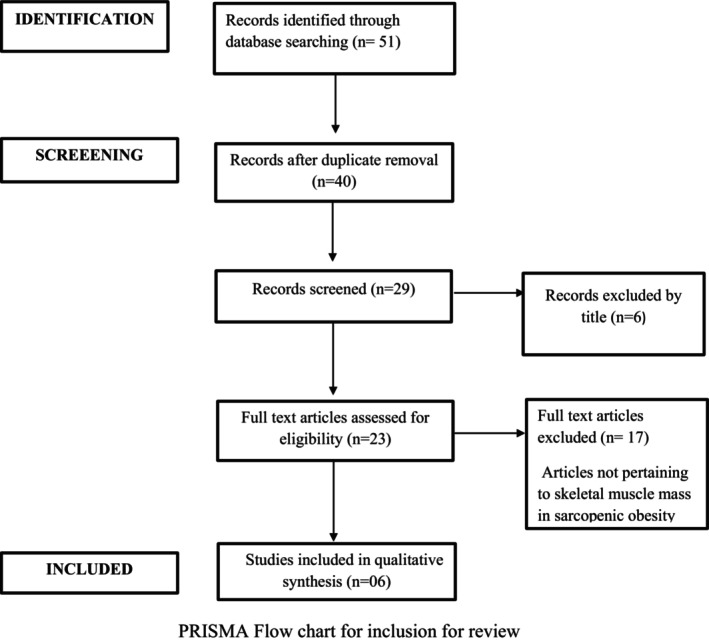
PRISMA chart. PRISMA, Preferred Reporting Items for Systematic Reviews and Meta‐Analyses.

The following search strategy was used: ((obesity and sarcopenia)) AND (exercise training)) AND (skeletal muscle mass)) AND (appendicular muscle mass)). Following the completion of the search, the references in the papers were chosen and examined in order to incorporate additional publications that were not found in the initial computerized search (Figure 1).

### 2.9 Focused question

The scoping review question was primarily focused on: What are the available evidences in the form of RCTs proving the effectiveness of delivering exercise as an intervention to sarcopenic obesity in older adult patients on muscle mass parameters?

### 2.10 Data extraction

Following the first evaluation of the received articles and identification of the key factor present in the studies, the next stage is to extract more data in order to produce the results. During the careful study of each article, we discovered that the exercise training methods comprised of aerobics in the form of walking and treadmill training and resistance equipment in the form of Kettle bells and elastic bands.

### 2.11 Data items

Individual data were acquired from available eligible RCTs. The characteristics considered were (title, author, and year of publication, number of samples, research duration, kind of exercise intervention, extraction procedure, and pre and post SMM and appendicular muscle mass [*P* value], and remarks/conclusion; Table 1).

**TABLE 1:** Available evidence depicting muscle mass parameter improvements as a result of exercise intervention

Available evidences	Sample size	Age group included	Duration of the study	Types of exercise intervention	Mode of exercise	*P* value <0.05	Remarks/conclusion
Hung‐Ting Chen et al, 2017, RCT	n = 93	65–75 y	12 wk	Combined exercises—aerobic + resistance exercise	Resistance—gym related training exercises Aerobic—stepping on the spot, knee lifts, high knee running, rowing arm swings, arm swings, twist steps, arm raises, squats, V steps, mambo steps, diamond steps, and point step jumps	0.05	RT, AT, or CT boosted muscle mass while decreasing total fat mass and visceral fat. The training groups, particularly the RT group, outperformed the control group in terms of muscle strength performance.
Hung‐Ting Chen et al, 2018, RCT	n = 33	65–75 y	12 wk	Resistance kettle bell training	Kettle bell swing, kettle bell deadlift, kettle bell goblet squat, squat lunge, two‐arm kettle bell military press, Turkish get up, and comprehensive dynamic workout	0.05	Kettle bell exercise improves sarcopenia index, grip strength, and back strength
Shan‐Shan Shen et al 2016, RCT	n = 35	Above 60 y	12 wk	Nutrition and combined exercise	Nutrition‐high‐quality protein intake Exercise group‐flexibility exercise, aerobic exercise, balance and progressive RT	0.05	Both nutrition and exercise interventions have been shown to be successful, and the combined interventions will improve sarcopenia more than either intervention alone
Karina S. S. Vasconcelos et al, 2016, RCT	n = 28	65–80 y	10 wk	Resistance exercise	Lower limb strength, power, and endurance, with open chain and closed kinetic chain exercises	0.01	There was no evidence of effectiveness in increasing physical function in older women with sarcopenic obesity
Chun‐De Liao et al, 2017, RCT	n = 46	65–75 y	12 wk	Elastic resistance exercise	Thera band—Progressive resistance exercise training	0.001	Elastic RT improved body composition, muscle indices, and physical function in sarcopenic patients with obesity
Jinkee Park et al, 2017, RCT	n = 50	70–80 y	24 wk	Combined exercises—aerobic + resistance exercise	Aerobic‐sideways and forward walking and slow and fast indoor walking. Resistance–gym related training exercises.	0.05	After a 24‐wk exercise training program, appendicular muscle mass increased

Abbreviations: AT, aerobic training; CT, combined training; RCT, randomized controlled trial; RT, resistance training.

## 3 DISCUSSION

The major goal of this scoping study was to determine the effectiveness of exercise interventions on muscle mass in older adults with sarcopenic obesity. The outcome measures used to determine muscle mass (particularly in SMM and ASM) varied among research.

The *P* values based on baseline (pre‐test) and post‐test values was used to measure effectiveness on muscle mass. Resistance workouts resulted in greater benefits than aerobic exercises. Resistance exercise, as opposed to aerobic exercise, which primarily works on cardiorespiratory fitness, directly works on muscle fibers, hence increasing strength.

According to Chen et al,^13^ the RT, AT, and combined training (CT) groups all had significantly higher SMM and ASM as well as lower BFM and ventricular fibrillation (VF) when compared to the control group (*P* < 0.05).

Chen et al^8^ discovered that kettle bell training significantly increases the sarcopenia index, grip strength, and back strength in the elderly with sarcopenia (*P* < 0.05).

Vasconcelos et al^4^ discovered that a progressive resistance exercise program with a high‐speed component was ineffective in increasing physical function in older women with sarcopenic obesity (*P* < 0.01).

According to Liao et al,^22^ resistance exercise training not only improves body composition in obese senior persons but also facilitates muscle mass gain, which may boost physical function by improving muscle strength (*P* < 0.001).

Park et al^5^ reported that a combined exercise program enhanced ASM in both the aerobic and resistance exercise groups of elderly women with sarcopenic obesity (*P* < 0.05).

Aerobic and resistance exercise both serve as preventative and therapeutic strategies. Any type of exercise training will boost protein synthesis, muscle mass, and muscular strength. According to Ozaki et al, aged people who regularly engage in moderately strenuous aerobic exercise had better maximum oxygen uptake, muscle growth, and function in their lower limbs. RT and aerobic exercise (AT), according to Sarsens et al, can both considerably reduce obesity‐related factors. Previous research found that aerobic exercise had a significant effect on lowering obesity risk factors, despite the fact that resistance exercise improved muscle mass and strength. Furthermore, this study found that weight training produces superior results than other types of exercise.

We researched and discovered that RT is more beneficial than other types of exercise in improving muscle mass in older persons with sarcopenic obesity.

### 3.1 Limitations

There were some limitations to this scoping review. We reviewed at RCTs to get *P* values so that the effectiveness of exercise interventions could be effectively explored. Furthermore, we were unable to consider other reviews and studies. We discovered the fewest number of research in the specified time period due to a lack of awareness of sarcopenic obesity among older persons, as seen by the gaps in exercise regimens.

## 4 CONCLUSION

Resistance exercise is more effective than other types of exercise in improving muscle mass in older persons with sarcopenic obesity, according to this scoping study. Further interventional research in the sarcopenic population can be done, and a definitive successful protocol can be designed and used globally to cure and minimize the occurrence of sarcopenia.

## AUTHOR CONTRIBUTIONS

Both Primary/Corresponding author and Secondary authors have equal contributions in the article.

## FUNDING INFORMATION

No funding was received for this study.

## CONFLICT OF INTEREST STATEMENT

Nothing to disclose.

## Data Availability

Data are openly available in a public repository that issues datasets with DOIs.
